# Astrocyte proliferation in the hippocampal dentate gyrus is suppressed across the lifespan of dystrophin‐deficient *mdx* mice

**DOI:** 10.1113/EP092150

**Published:** 2025-01-10

**Authors:** Kimberley A. Stephenson, Polly Peters, Mark G. Rae, Dervla O'Malley

**Affiliations:** ^1^ Department of Physiology, School of Medicine University College Cork Cork Ireland

**Keywords:** astrocytes, dentate gyrus, Duchenne muscular dystrophy, dystrophin, microglia, neurogenesis

## Abstract

Absence of the structural protein, dystrophin, results in the neuromuscular disorder Duchenne Muscular Dystrophy (DMD). In addition to progressive skeletal muscle dysfunction, this multisystemic disorder can also result in cognitive deficits and behavioural changes that are likely to be consequences of dystrophin loss from central neurons and astrocytes. Dystrophin‐deficient *mdx* mice exhibit decreases in grey matter volume in the hippocampus, the brain region that encodes and consolidates memories, and this is exacerbated with ageing. To understand changes in cellular composition that might underpin these age‐related developments, we have compared neurogenesis and the prevalence of immunofluorescently identified newly born and mature neurons, astrocytes and microglia in the dentate gyrus of *mdx* and wild‐type mice at 2, 4, 8 and 16 months of age. The number of adult‐born neurons was suppressed in the dentate gyrus subgranular zone of 2‐month‐old *mdx* mice. However, the numbers of granule cells and GABA_A_ receptor, alpha 1‐expressing cells were similar in wild‐type and *mdx* mice at all ages. Strikingly, the numbers of astrocytes, particularly in the dentate gyrus molecular layer, were suppressed in *mdx* mice at all time points. Thus, dystrophin loss was associated with reduced hippocampal neurogenesis in early life but did not impact the prevalence of mature neurons across the lifespan of *mdx* mice. In contrast, normal age‐related dentate gyrus astrocyte proliferation was suppressed in dystrophic mice. Astrocytes are the most abundant cell type in the brain and are crucial in supporting neuronal function, such that loss of these cells is likely to contribute to hippocampal dysfunction reported in *mdx* mice.

## Abstract (highlights)

- **What is the central question of this study?**
  Cognitive deficits are associated with the loss of the structural protein, dystrophin, from neurons and glia. To determine whether alterations in the cellular make‐up of the dentate gyrus underlie age‐related functional deterioration, we examined dystrophic *mdx* mice at four different ages.
- **What is the main finding and its importance?**
  Although neurogenesis was suppressed in young *mdx* mice, the number of mature dentate gyrus principal cells was stable. Astrocyte numbers were suppressed at all ages in *mdx* mice. Loss of astrocytes, the most abundant cell type in the brain, which are crucial in supporting neuronal function, might be a key player in hippocampal dysfunction associated with dystrophin loss.

## 1 INTRODUCTION

Individuals with Duchenne Muscular Dystrophy (DMD), an X‐linked disorder detected in 1 in every 3500 male births, exhibit progressive muscular atrophy, loss of mobility (McDonald et al., 2018) and limited life expectancy (Yiu & Kornberg, 2008). This disease is caused by mutations in the dystrophin gene, resulting in loss of the sarcolemma‐spanning structural protein, dystrophin, from skeletal muscle fibres, making them susceptible to contraction‐induced damage (Lynch, 2004), inflammation and myophagocytosis (Tulangekar & Sztal, 2021). However, DMD is a multisystem disorder, because dystrophin is also expressed in cardiac and smooth muscle, endocrine glands and neurons. In the brain, dystrophin is restricted to specific regions, including the hippocampus, cerebral cortex, amygdala and cerebellum (Knuesel et al., 2000; Lidov et al., 1993, 1990; Sekiguchi et al., 2009), but is most highly expressed in the hippocampus and amygdala (Doorenweerd et al., 2017). With tissue‐specific dystrophin promoters for brain and skeletal muscle cells (Blake et al., 2002), there is significant variability in the protein products generated in different tissues. In the brain, Dp427, the full‐length dystrophin isoform, is found only in neurons, mostly in the postsynaptic density, co‐expressed with GABA_A_ receptors (Anderson et al., 2002; Knuesel et al., 1999). Dp71 is ubiquitous in the brain, particularly in astrocytes (Haenggi et al., 2004; Tadayoni et al., 2012) but also detected in neuronal cells in the dentate gyrus (DG) (Gorecki & Barnard, 1995). In astrocytes derived from human pluripotent stem cells from individuals with DMD, astrocyte morphology and function were defective, which appeared to contribute to neural impairment (Lange et al., 2022).

The functional relevance of dystrophin in the CNS is evidenced by the increased prevalence of neuropsychiatric symptoms and deficits in cognitive function in individuals with DMD (Rae & O'Malley, 2016). This includes intelligence quotients that are 1 SD below the normal range (Ackerman et al., 1996; Felisari et al., 2000; Nardes et al., 2012) and poor verbal, short‐term and working memory (Hinton et al., 2000, 2001; Snow et al., 2013). In *mdx* mice, which lack dystrophin (Bulfield et al., 1984), the capacity to learn new tasks and store spatial memories is impaired (Chaussenot et al., 2015; Lopez et al., 2018; Vaillend et al., 2004, 1995), and long‐term potentiation, the molecular correlate of learning and memory consolidation, is blunted (Moore et al., 2002). Hippocampus‐dependent spatial learning and memory deteriorate with age in *mdx* mice (Bagdatlioglu et al., 2020), and age‐related cerebral atrophy in human DMD (Yoshioka et al., 1980) is indicative of progressive cognitive degeneration.

Orchestration of spatially and temporally regulated neural networks that encode and consolidate sensory experiences into persistent long‐term memories underpin hippocampal function (Lidov et al., 1993, 1990; Sekiguchi et al., 2009). The generation of differentiated neurons from neural stem cells in the subgranular zone (SGZ) of the DG, which then integrate into existing neural pathways, is an important contributor to hippocampal function (Abbott & Nigussie, 2020). Dystrophin is present in mouse neural stem cells (Romo‐Yanez et al., 2020), and neurogenesis is disrupted in dystrophic *mdx* mice, although that might be secondary to a loss of peripheral muscle neuronal nitric oxide synthase (Deng et al., 2009). Neural stem cells can differentiate into glial cells and neurons in the adult brain (Alvarez‐Buylla et al., 2001; Gebara et al., 2016; Kriegstein & Alvarez‐Buylla, 2009), with astrocytes supporting neuronal survival, migration and integration (Alvarez‐Buylla et al., 2001; Sultan et al., 2015). Moreover, communication between astrocytes and neurons contributes to the recruitment of new neurons into pre‐existing neural circuits (Bezzi et al., 2001).

Several studies in human DMD patients (al‐Qudah et al., 1990; Bresolin et al., 1994; Rae et al., 1998) and in *mdx* mice (Bulfield et al., 1984; Miranda et al., 2009; Yoshihara et al., 2003) reported no gross abnormalities to brain structure. However, in more recent studies, enlargement of the lateral ventricles was apparent in older *mdx* mice (∼9–18 months old), possibly attributable to grey matter atrophy (Bagdatlioglu et al., 2020; Xu et al., 2015). In young (2‐ to 3‐month‐old) mice, changes in the density, size and/or shape of cells in cortical regions and the brainstem were observed (Carretta et al., 2004, 2001; Minciacchi et al., 2010; Sbriccoli et al., 1995). The aim of this study was to compare neurogenesis and relative numbers of differentiated neurons, astrocytes and microglia in the DG region of the hippocampus of dystrophic *mdx* and wild‐type (WT) mice at 2, 4, 8 and 16 months of age.

## 2 MATERIALS AND METHODS

### 2.1 Ethical approval

All animal experiments were approved and performed following guidelines set out by the HPRA (Health Products Regulatory Authority), Ireland and following project authorization (AEI9130/P088), in addition to individual authorization (AEI9130/I303). Animals were euthanized in accordance with European Directive 2010/63/EU.

### 2.2 Animals and tissue collection

Dystrophin‐deficient C57BL/10ScSn‐Dmd*^mdx^*/J (*mdx*, dystrophic) and C57BL/10ScSn and WT controls, purchased from Jackson Laboratories (Bar Harbor, ME, USA), were used for these studies. Breeding colonies of both *mdx* and WT mice were established and maintained in the Specific Pathogen Free (SPF) facility, Biological Services Unit, University College Cork, Ireland. Weaned mice were housed in individually ventilated cages with environmental enrichment and kept under an artificial light–dark cycle provided with light between 06:00 and 18:00 h, with free access to drinking water and standard chow. Hemizygotic male mice were used for all studies.

Mice were euthanized with isoflurane overdose and subsequently perfused with intracardiac saline (15 mL), followed by 15 mL formalin (Sigma‐Aldrich, St Louis, MO, USA). Whole brains were excised from the skull and post‐fixed in formalin (Sigma‐Aldrich) for 72 h, then cryo‐protected by immersion in a 30% sucrose solution (Sigma‐Aldrich) made up in 0.1 mM PBS. Samples were frozen in isopentane and stored at −80°C.

### 2.3 Hippocampal slice preparation

Coronal sections (20 µm sections) from the anterodorsal hippocampus (bregma −1.655 to −2.255) were cryo‐sectioned (Leica, Germany) in optimal cutting temperature (OCT) embedding medium (Thermofisher Scientific, MA, USA). Approximately 20 sections were taken from each hippocampus, and staining was compared between WT and *mdx* mice on matched sections. Cryo‐sectioned tissue was mounted onto positively charged glass slides (SuperFrost Plus, VWR International, Radnor, PA, USA) before immunofluorescence staining. Hippocampus‐containing whole brain slices from at least three WT and *mdx* mice were compared for each of the immunopositive cell markers used.

### 2.4 Immunofluorescence and confocal microscopy

Fixed brain hemisphere sections (20 µm thick) mounted on glass slides were permeabilized with 0.1% Triton X‐100 and blocked with 1% donkey serum (Sigma Aldrich, UK). Taking every fifth section, hippocampus‐containing brain slices were immunolabelled with primary antibodies against doublecortin (DCX, rabbit, 1:100; Invitrogen: Cat. no.: 48‐1200), NeuN (rabbit, 1:100; Invitrogen: Cat. no.: PA5‐78499), glial fibrillary acidic protein (GFAP; rabbit, 1:100; Invitrogen: Cat. no.: PA5‐16291), anti‐GABA_A_ receptor, alpha 1 (1:100, mouse; Abcam: Cat. no. ab94585, Cambridge, UK) and TMEM119 (rabbit, 1:500; Atlas Antibodies, Sweden), respectively. Appropriate TRITC (red staining) and FITC (green staining) fluorophores (1:250, 2 h, 37°C; Jackson Immunoresearch, PA, USA) were used to visualize the primary antibody staining. Tissue sections were mounted using Dako‐fluorescent mounting medium containing 4′,6‐diamidino‐2‐phenylindole (DAPI; cyan/blue staining; Agilent Pathology Solutions Santa Clara, CA, USA) to visualize cellular nuclei and covered with a glass coverslip.

Images were captured using a FVl0i Olympus confocal microscope with Fluoview software (FV10i‐SW; Olympus Europe, Hamburg, Germany) or an Olympus BX51 microscope with an Olympus DP71 camera. Cell Sens (Olympus Europe) was used to capture digital images of the whole hippocampus. The laser and saturation settings were set at the beginning and kept constant for every immunofluorescently labelled protein while imaging all the samples within a group. No non‐specific fluorescent immunotagging was detected in negative control experiments, where tissues were incubated with primary antibodies in the absence of secondary antibodies or with secondary antibodies alone.

### 2.5 Cell counting and analysis

Using a sampling scheme similar to that described previously (West et al., 1991), the number of antibody‐identified cell types in the DG region (including the granule cell layer, hilus and subgranular zone) was calculated by counting the number of cells, satisfying specific cell‐type criteria, per field using ImageJ software (NIH, USA, https://imagej.net/ij/). A template containing three fields of fixed area was overlaid on the immunofluorescence images. A mean cell count was taken from the three regions selected. Although there are limitations to this strategy, the criteria were applied rigidly to all samples. At least two researchers (both blinded to the groups) counted the cells, and an average value was taken from each count.

### 2.6 Statistics

The data are represented as mean values ± SD. Statistical analysis was conducted on mean values from several images taken from both hippocampal hemispheres, with one animal taken as *n* = 1. Two‐way ANOVAs with Sidak's multiple comparison tests or Student's two‐tailed unpaired *t*‐tests were used, as appropriate (GraphPad Prism v.8.4.2). A value of *P *≤ 0.05 was considered significant.

## 3 RESULTS

### 3.1 Neurogenesis in the DG is prematurely suppressed in *mdx* mice

**FIGURE 1:**
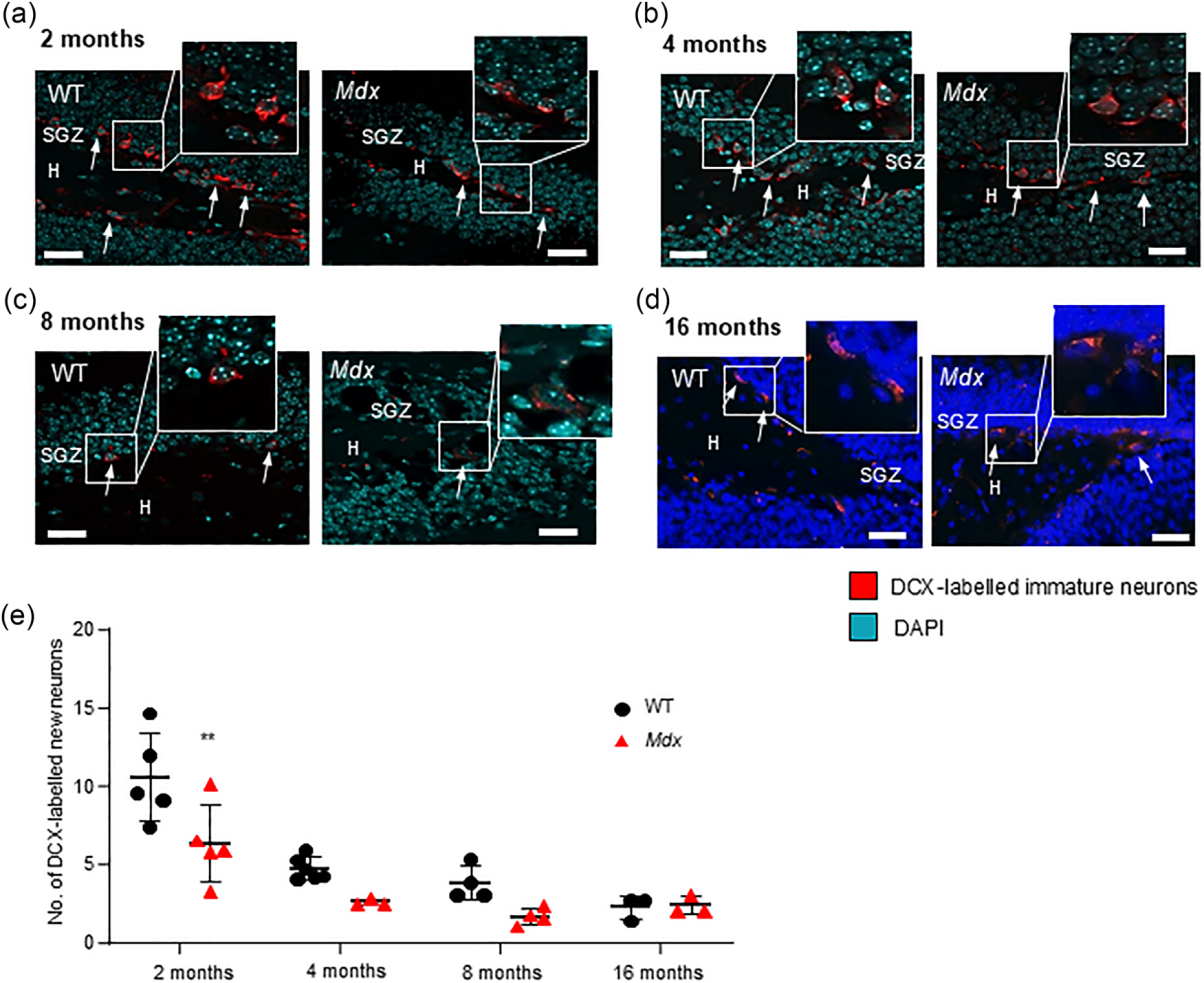
Neurogenesis is suppressed in the DG of young dystrophin‐deficient *mdx* mice. The representative immunofluorescence images show DCX‐labelled migrating neurons (red staining) in the hippocampal DG of WT and *mdx* mice aged 2 (a), 4 (b), 8 (c) and 16 months (d). Co‐staining with DAPI (cyan or blue staining) was used to visualize the granule cell body layer. Insets show digitally magnified images of immunolabelled new neurons. Scale bars: 35 µm. (e) The data plot illustrates the pooled average data for the number of DCX‐positive neurons per field in WT (black dots) and *mdx* (red triangles) mice at each time point. **P < 0.01. Abbreviations: DCX, doublecortin; DG, dentate gyrus; H, hilus; SGZ, subgranular zone; WT, wild‐type.

Neurogenesis in the hippocampal DG region results in adult‐born neurons that can integrate into existing neuronal circuits (Gage, 2000) and contribute to neuronal plasticity. An antibody against DCX was used as a marker of neurogenesis (Couillard‐Despres et al., 2005), because it is associated with migration, axonal guidance and dendrite sprouting. Similar to previous work (Klempin et al., 2011), DCX‐labelled cells were detected in the SGZ (Figure 1a–d; red‐stained cells indicated by arrows), on the innermost margins of the DG granule cell body layer (illustrated by blue DAPI staining) from WT and *mdx* mice at 2, 4, 8 and 16 months of age. Consistent with decreased neurogenesis in ageing (Mathews et al., 2017), a strong effect of time was detected [*F*(3,25) = 27.1, *P *< 0.0001, two‐way ANOVA] in both WT and dystrophic *mdx* mice. However, a difference between WT and *mdx* mice was also apparent [*F*(1,25) = 13.01, *P* = 0.001], with a significant decrease in the number of DCX‐positive cells in 2‐month‐old *mdx* mice [6.31 ± 2.5, *n* = 5 mice (64 images)] compared with WT mice [10.55 ± 2.8, *n* = 5 mice (35 images); *P* = 0.001; Figure 1a, e]. No significant difference in neurogenesis was evident at 4 months [*n* = 3 WT (70 images) and *n* = 3 *mdx* (42 images) mice; *P* = 0.27; Figure 1b, e], 8 months [*n* = 3 WT (21 images) and *n* = 4 *mdx* (11 images) mice; *P* = 0.26; Figure 1c, e] or 16 months [*n* = 3 WT (9 images) and *n* = 3 *mdx* (10 images) mice; *P* > 0.99; Figure 1d, e]. These data suggest that age‐related decreases in hippocampal neurogenesis are accelerated in dystrophic hippocampal tissue.

### 3.2 Mature DG granule cells were comparable in WT and *mdx* mice at different age points

**FIGURE 2:**
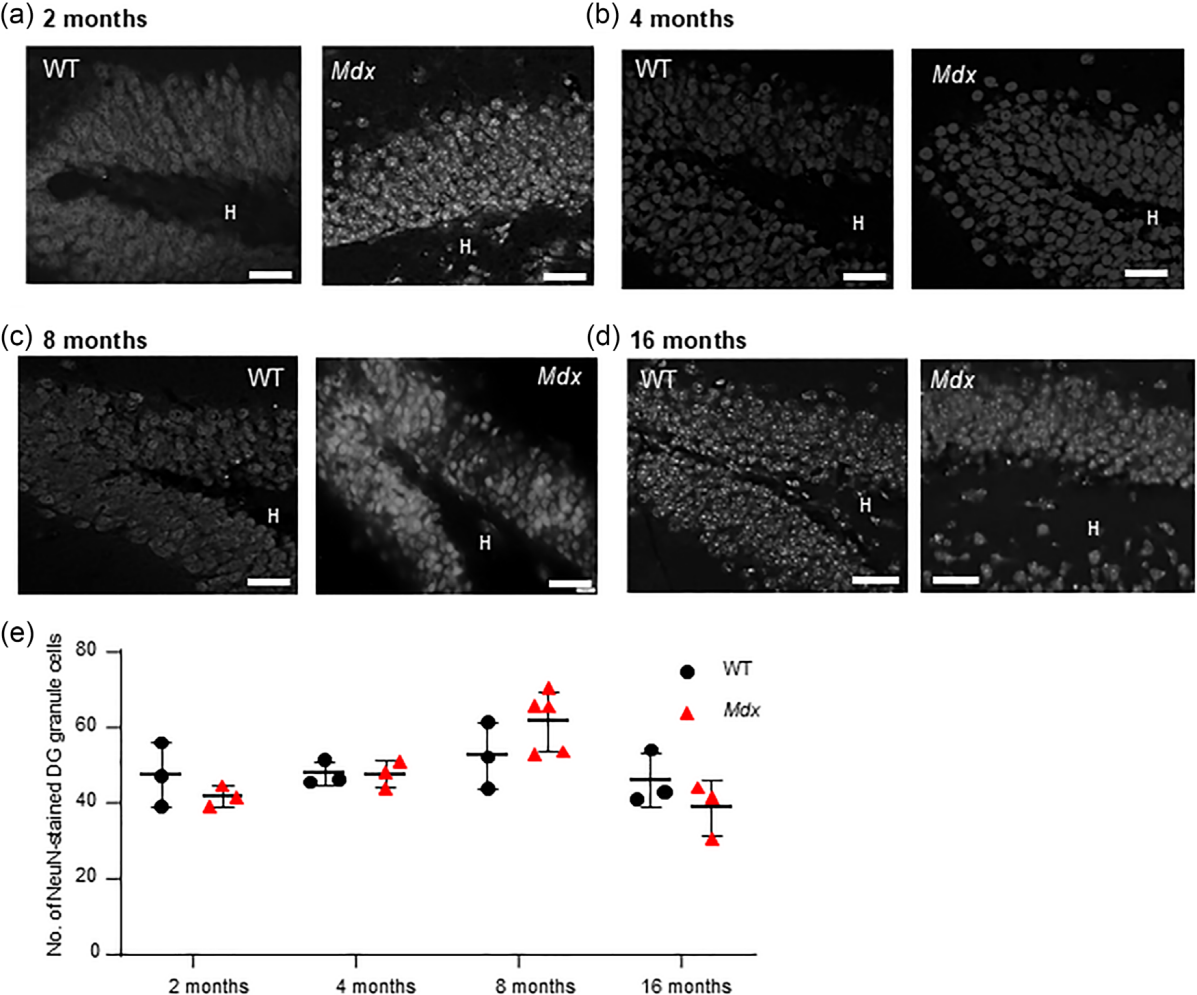
The density of granule cells is comparable between WT and *mdx* mice at different ages. The images show mature neuronal cell bodies labelled with the pa‐neuronal marker, NeuN, which labels neuron‐ specific nuclear protein in the granular cell layer of WT and *mdx* hippocampal dentate gyrus at 2 (a), 4 (b), 8 (c) and 16 months of age (d). Scale bars: 35 µm. (e) The data plot illustrates the averaged pooled data for the number of NeuN‐stained mature neurons in the granule cell body layer per field in WT (black dots) and *mdx* (red triangles) mice at each time point. Abbreviations: H, hilus; WT, wild‐type.

Neural progenitor cells migrate into the hippocampal granule cell layer and differentiate into neurons or glial cells (Ming & Song, 2011). In the granule cell body layer of the hippocampal DG, the pan‐neuronal marker, NeuN, which labels neuron‐specific nuclear protein, was used to identify mature neurons in WT and *mdx* hippocampal slices. The numbers of neurons per field (overlay of defined area) were compared between strains. In 2‐month‐old mice, no difference in the density of granule cell bodies was detected between WT (*n* = 24 fields from 3 mice) and *mdx* mice (*n* = 20 fields from 3 mice, *P* = 0.79; Figure 2a, e). Likewise, 4‐month‐old WT (*n* = 51 fields from 3 mice) and *mdx* (*n* = 36 fields from 3 mice, *P* > 0.99; Figure 2b, e), 8‐month‐old WT (*n* = 35 fields from 3 mice) and *mdx* (*n* = 39 fields from 3 mice, *P* = 0.29; Figure 2c, e), and 16‐month‐old WT (*n* = 28 fields from 3 mice) and *mdx* (*n* = 31 fields from 3 mice, *P* = 0.58; Figure 2d, e) mice were all comparable. Thus, across all age points, no difference between strains was evident [*F*(1,18) = 0.37, *P* = 0.72, two‐way ANOVA]. An effect of age on the density of granule cells was detected [*F*(3,18) = 6.4, *P* = 0.004], but no interaction between factors. To support these findings, we also counted DAPI‐labelled cells in the granule cell layer. No difference in the number of DAPI‐stained cell bodies was detected between strains [*F*(1,24) = 0.37, *P* = 0.55, two‐way ANOVA]. However, an effect of age was apparent [*F*(3,24) = 58.5, *P* < 0.0001], resulting in an interaction between strain and age [*F*(3,24) = 5.5, *P* = 0.005, data not shown]. These data support the idea that the density of excitatory granule cells is maintained across the lifespan of mice that lack dystrophin.

### 3.3 GABA_A_ receptor, alpha 1‐labelled non‐principal cells were comparable in WT and *mdx* mice at different age points

**FIGURE 3:**
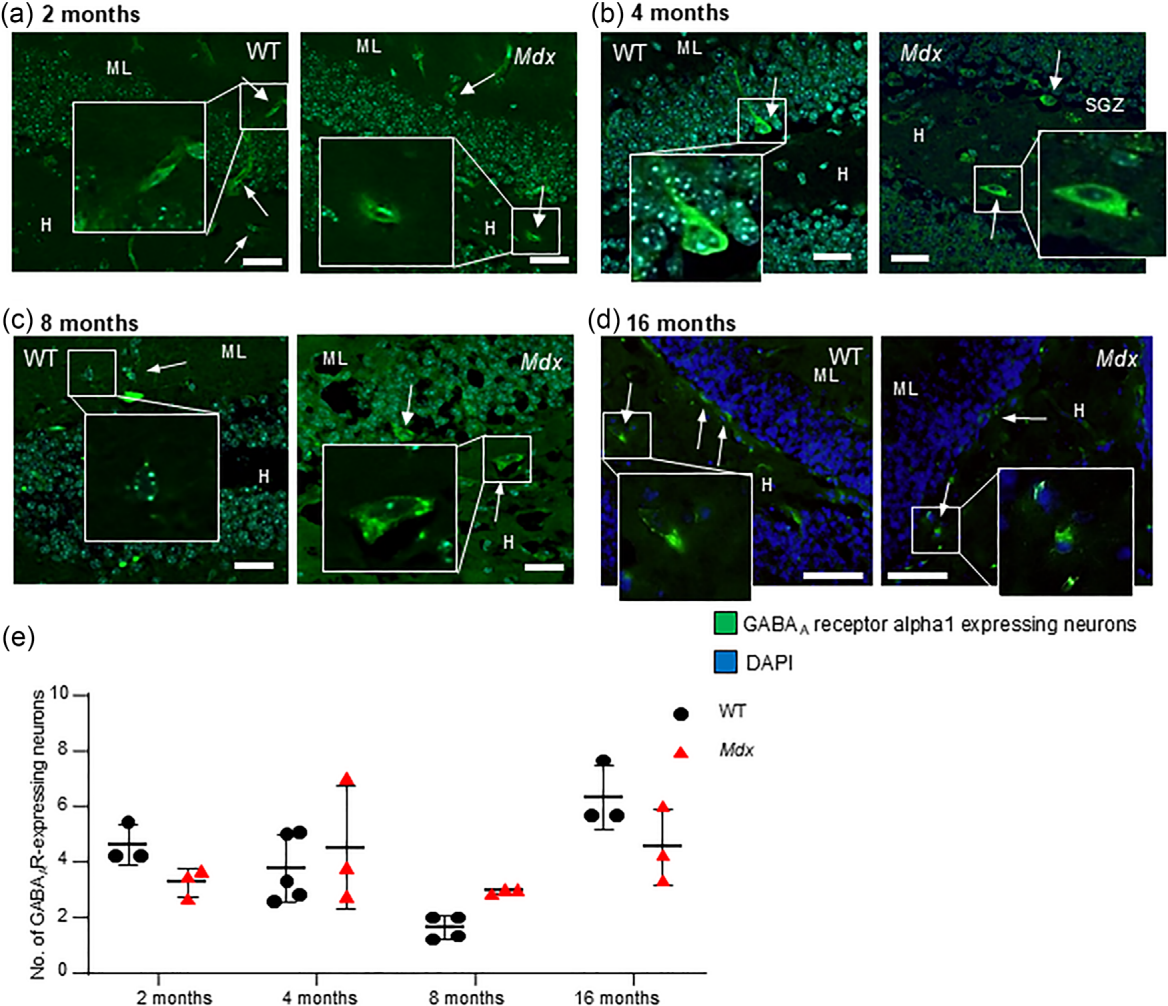
The number of GABA_A_ receptor‐expressing neurons in the DG is similar in WT and *mdx* mice at all ages examined. The representative immunofluorescence images illustrate GABA_A_ receptor‐expressing inhibitory interneurons (green staining; arrows) within the DG co‐labelled with a DAPI nuclear stain (cyan/blue staining) in 2 (a), 4 (b), 8 (c) and 16‐month‐old (d) WT and *mdx* hippocampal slices. Insets show digitally magnified images of immunolabelled inhibitory neurons. Scale bars: 35 µm. (e) The data plot illustrates the averaged pooled data for the number of GABA_A_ receptor‐expressing neurons per field in WT (black dots) and *mdx* (red triangles) mice at each time point. Abbreviations: DG, dentate gyrus; H, hilus; ML, molecular layer; WT, wild‐type.

**FIGURE 4:**
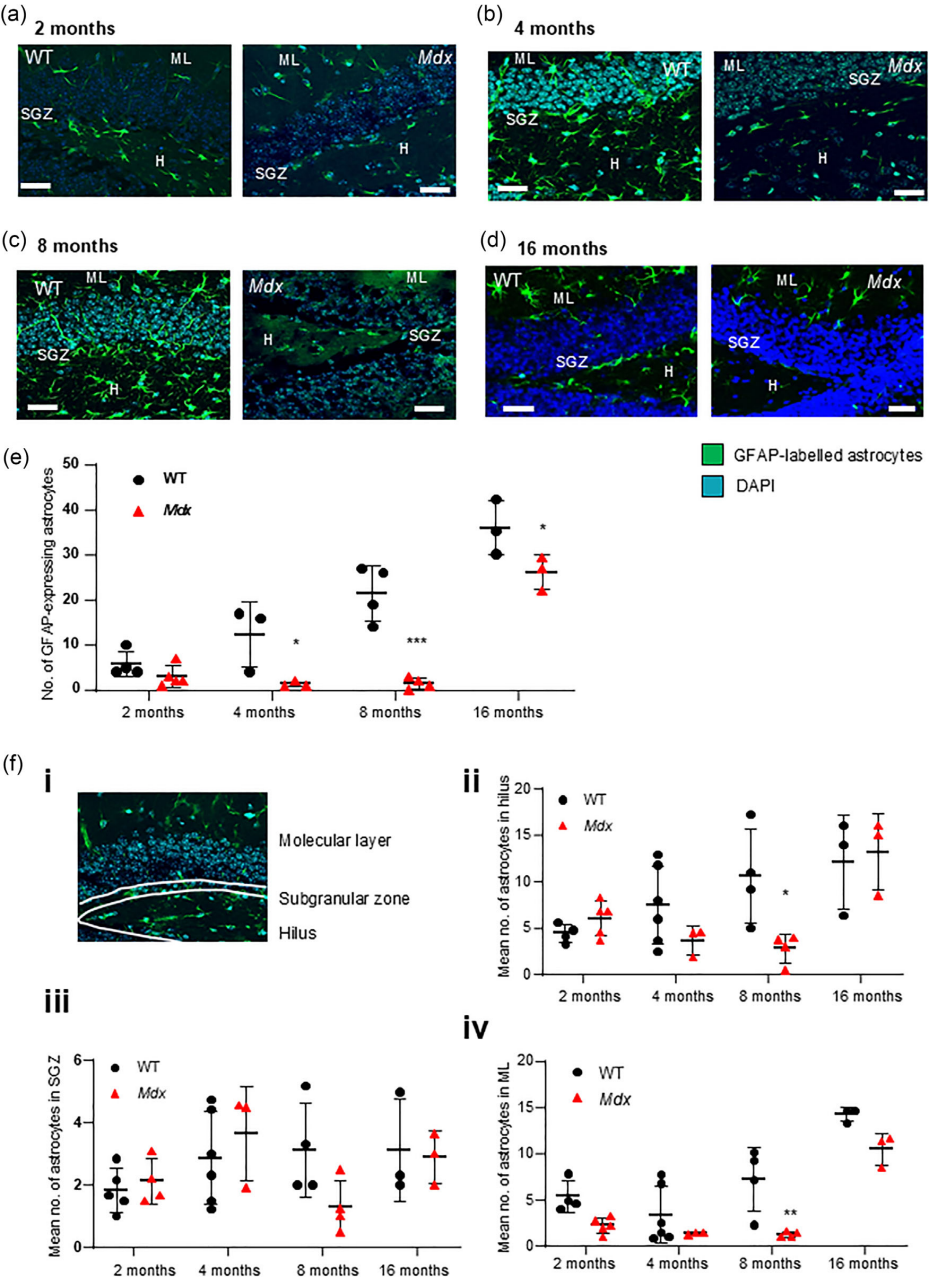
Age‐related increases in DG astrocyte numbers are suppressed in *mdx* mice. The representative immunofluorescent images illustrate GFAP‐immunotagged astrocytes in the hippocampal DG from WT and *mdx* mice at 2 (a), 4 (b), 8 (c) and 16 months of age (d). The images show astrocytes (green staining) and the granule cell body layer made visible with DAPI staining (cyan/blue staining). Scale bars: 35 µm. (e) The data plot illustrates the pooled averaged data for the number of GFAP‐stained astrocytes per field in WT (black dots) and *mdx* (red triangles) mice at each time point. [f(i)] The representative image of the DG stained for GFAP and DAPI illustrates the distinct regions where astrocyte numbers were monitored. [f(ii–iv)] The data plots illustrate the average number of astrocytes per field in WT and *mdx* mice at each time point in the hilus (ii), the subgranular zone (iii) and the molecular layer (iv). *P < 0.05 and ***P < 0.001. Abbreviations: DG, dentate gyrus; GFAP, glial fibrillary acidic protein; H, hilus; ML, molecular layer; SGZ, subgranular zone; WT, wild‐type.

Given the importance of GABA in the generation and integration of new hippocampal neurons (Wang et al., 2005), disruption of GABAergic regulation of hippocampal function as a consequence of dystrophin loss (Vaillend et al., 2010) might be an important factor contributing to decreased neurogenesis in the early life of *mdx* mice. We examined the prevalence of cells immunopositive for GABA_A_ receptor, alpha 1 in the DG of WT and *mdx* mice. Consistent with other reports (del Tongo et al., 2009), GABA_A_ receptor, alpha 1 was expressed non‐uniformly in the cells of the DG. Higher levels of expression were detected in immunopositive cells with large, often pyramidal‐shaped cell bodies, primarily located in the hilus and amongst the smaller principal cell bodies in the granule cell layer of the DG. No differences between the numbers of these GABA_A_ receptor, alpha 1 subunit‐expressing cells were detected in 2‐month‐old WT (*n* = 39 images from 3 mice) and *mdx* (*n* = 34 images from 3 mice, *P* = 0.49; Figure 3a, e) or 4‐month‐old WT (*n* = 47 images from 5 mice) and *mdx* (*n* = 27 images from 3 mice, *P* = 0.84; Figure 3b, e) mice. This trend continued in 8‐month‐old (WT, *n* = 15 images from 3 mice vs. *mdx*, *n* = 14 images from 3 mice, *P* = 0.46; Figure 3c, e) and 16‐month‐old mice (WT, *n* = 9 images from 3 mice vs. *mdx*, *n* = 10 images from 3 mice, *P* = 0.23; Figure 3d, e). Thus, there was no difference between strains in the expression of DG cells immunopositive for GABA_A_ receptor, alpha 1 subunits [*F*(1,19) = 0.38, *P* = 0.55]. Overall, an effect of ageing was detected [*F*(3,19) = 8.45, *P* = 0.0009], but the interaction between factors did not reach significance [*F*(3,19) = 3.04, *P* = 0.06].

### 3.4 Age‐related increase in astrocyte numbers is suppressed in *mdx* mice

Striking differences between mouse strains in the overall number of GFAP‐immunolabelled astrocytes were noted at different ages. In 2‐month‐old mice, the number of GFAP‐stained astrocytes per field was similar in WT (*n* = 19 fields from 4 mice) and *mdx* (*n* = 41 fields from 5 mice, *P* = 0.81; Figure 4a, e] mice. By 4 months, however, astrocyte numbers had increased in WT mice (*n* = 30 fields from 3 mice) but not in *mdx* mice (*n* = 39 fields from 3 mice, *P* = 0.02; Figure 4b, e). This pattern continued in older 8‐month‐old mice, with a further increase in WT mice (*n* = 20 fields from 4 mice), but much lower numbers in *mdx* DG tissue (*n* = 15 fields from 4 mice, *P *< 0.0001; Figure 4c, e). In aged, 16‐month‐old mice, a delayed increase in astrocyte numbers was apparent in *mdx* mice (*n* = 8 fields from 3 mice), but prevalence remained lower than in WT mice (*n* = 9 fields from 3 mice, *P* = 0.04; Figure 4d, e). A strong effect of age was detected by two‐way ANOVA [*F*(3,21) = 53.82, *P *< 0.0001], with distinct differences between strains [*F*(1,21) = 46.97, *P *< 0.0001] leading to an interaction between strain and age (*F*(3,21) = 5.92, *P* = 0.004].

Astrocyte function is intimately tied to its location, and glial heterogeneity in the DG region has implications for the formation of neural circuits (Karpf et al., 2022). We therefore explored the prevalence of GFAP‐labelled glial cells in the polymorphic layer of the hilus, the SGZ, the site of neurogenesis and the granule cell body layer into the molecular layer [Figure 4f(i)] to determine whether the observed decrease in prevalence of DG astrocytes in *mdx* mice was specific to any one of these regions. Reflecting variability in functions (Chalmers et al., 2024), astrocyte morphology differed between regions. In the *mdx* hilus, a significant effect of age was detected [*F*(3,24) = 6.65, *P* = 0.002]. Despite suppression of astrocytes at 8 months (*P* = 0.05), overall differences between strains were not significant in this region [*F*(1,24) = 3.24, *P* = 0.08]. However, there was an interaction between strain and age [*F*(3,24) = 3.27, *P* = 0.04; Figure 4f(ii)]. In the SGZ, the number of astrocytes was comparable at all time points, with no differences evident between strains or at different ages [Figure 4f(iii)]. However, in the ML, a region that contains the dendritic branches of the granule cell bodies, astrocyte numbers were decreased at 8 months (*P* = 0.002). A clear strain effect was evident [*F*(1,24) = 23.87, *P* < 0.001], as was the impact of mouse age [*F*(3,24) = 30.38, *P* < 0.001]. However, no interaction between factors was detected [Figure 4f(iv)].

### 3.5 Number of active microglia was increased only in 2‐month‐old *mdx* mice

**FIGURE 5:**
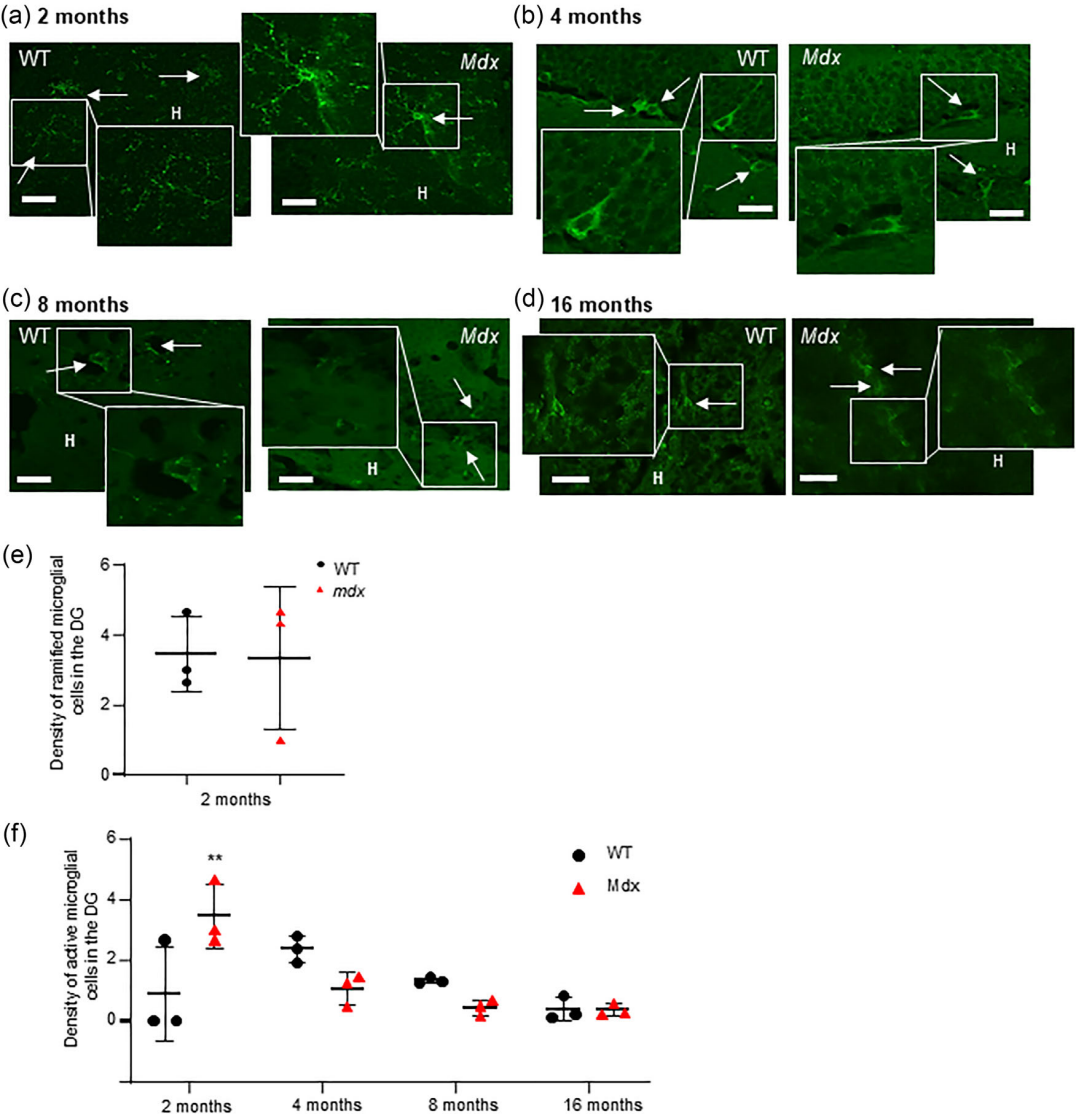
Active DG microglia are increased in 2‐month‐old *mdx* mice. The representative immunofluorescent images illustrate TMEM119‐immunotagged microglial cells in the hippocampal DG from WT and *mdx* mice at 2 (a), 4 (b), 8 (c) and 16 months of age (d). Insets show digitally magnified images of immunolabelled ramified microglia in the hippocampal dentate gyrus in 2‐month‐old tissue and active microglia in samples from older mice. Scale bars: 35 µm. (e) The data plot illustrates the pooled averaged data for the number of ramified microglia per field in WT (black dots) and *mdx* (red triangles) mice at 2 months old. (f) The data plot illustrates the pooled averaged data for the number of active microglia per field in WT and *mdx* mice at 2, 4, 8 and 16 months. **P < 0.01. Abbreviations: DG, dentate gyrus; H, hilus; WT, wild‐type.

Microglia can exist either as resting ramified immune cells or in an active state, which is characterized by an intermediate ‘bushy’ cell morphology. TMEM119‐labelled microglia with highly ramified morphology, small cell bodies and lots of branching, consistent with a resting, monitoring morphology, were detected primarily in the hilus region of the DG at similar densities in 2‐month‐old WT (*n* = 27 fields from 3 mice) and *mdx* (*n* = 18 fields from 3 mice, *P* = 0.94, Student's unpaired *t*‐test) mice (Figure 5a insets, e). However, in 2‐month‐old mice, TMEM119‐positive cells with morphology consistent with activation (increased size of the cell body, with retraction of processes resulting in an intermediate ‘bushy’ morphology that progresses towards an ‘amoeboid’ cellular shape) were elevated in *mdx* (*n* = 18 fields from 3 mice) compared with WT (*n* = 27 fields from 3 mice, *P* = 0.002; Figure 5f) mice. From 4 months onwards, TMEM119‐positive cells with ‘bushy’, active morphology were predominant in the DG (Figure 5b–d), with very few ramified microglia observed in either strain. The number of active microglia in 4‐month‐old WT mice (*n* = 46 fields from 3 mice) was similar to *mdx* mice (*n* = 40 fields from 3 mice, *P* = 0.16; Figure 5b, f). Likewise, at 8 months, the number of active microglia in WT (*n* = 32 fields from 3 mice) was not different from *mdx* (*n* = 38 fields from 3 mice, *P* = 0.49; Figure 5c, f) mice, and at 16 months, the number of active microglia in WT mice (*n* = 24 fields from 3 mice) was comparable to the number in *mdx* mice (*n* = 36 fields from 3 mice, *P* > 0.99; Figure 5d, f). Overall, an effect of age was detected by two‐way ANOVA [*F*(3,16) = 7.3, *P* = 0.003], but this was not different between strains [*F*(1,16) = 0.07, *P* = 0.79]. An interaction between factors [*F*(3,16) = 8.43, *P* = 0.0014] was detected.

## 4 DISCUSSION

In human DMD, increased prevalence of cognitive deficits (Ackerman et al., 1996; Felisari et al., 2000; Hinton et al., 2000, 2001; Nardes et al., 2012; Rae & O'Malley, 2016; Snow et al., 2013) implicates dystrophin in physiological processes underpinning learning and other behaviours. Indeed, the capacity to learn new tasks and store spatial memories is impaired in *mdx* mice (Chaussenot et al., 2015; Vaillend et al., 2004, 1995) and other dystrophin‐deficient rodent models (Hayward et al., 2022). Changes in the cellular make‐up and communication between different cell types in the hippocampus, the brain region critical for the learning and the encoding and consolidation of memories, might contribute to the manifestation of these deficits. The DG is the sensory input zone of the hippocampus. It regulates the flow of information and has a layered cellular structure. Excitatory granule cells are orientated such that the cell bodies form the granule cell layer. Dendrites extend into the molecular layer, and the neuronal axons project from the hilus to the CA3 region, forming the mossy fibre pathway. Inhibitory GABAergic interneurons provide fine‐tuned inhibitory regulation of granule cells. In the absence of dystrophin, others have reported reduced density of pyramidal neurons in the CA1 hippocampal region of 8‐month‐old *mdx* mice and suggested that this could be attributable to dysfunctional neurogenesis (Miranda et al., 2016). In our study, we explored changes in hippocampal cellularity in *mdx* mice early in disease development (2 months), at two age points of established disease (4 and 8 months) and in advanced disease (16 months).

We observed fewer DCX‐expressing newly born adult cells across the four time points examined in the lifespan of the *mdx* mouse; however, this reduction was significant only at the earliest time point (2 months). Overall numbers of excitatory granule cells did not differ between strains, and there was no effect of ageing observed. Likewise, loss of dystrophin did not modify the numbers of GABA_A_ receptor, alpha 1 subunit‐expressing non‐principal cells, with no change over time. The greatest impact of dystrophin loss was noted in astrocyte numbers. Continual astrogenesis over the lifetime of a mammal results in increased numbers of astrocytes, which we observed in WT mice. However, age‐related increases in astrocyte numbers were suppressed in dystrophin‐deficient *mdx* mice. This was particularly evident in the molecular layer and hilus regions. Finally, we explored whether the loss of dystrophin impacted the prevalence of microglia, in both the resting and active states. Numbers of active glia were increased in 2‐month‐old *mdx* mice, but no differences were detected later in life. Thus, although different isoforms of dystrophin are expressed in neurons and astrocytes, the greatest impact of the premature stop codon in exon 23 of the dystrophin gene in the *mdx* DG formation was on astrocyte numbers. Throughout the lifespan of *mdx* mice, the prevalence of these highly heterogeneous support cells, which are sensitive to changes in neuronal activity and can actively modulate synaptic plasticity, was suppressed. Given that astrocytes are the most abundant cell type in the CNS and are critical to normal neuronal function and survival, absent or dysfunctional astrocytes (Lange et al., 2022) are likely to be important players in cognitive deficits prevalent in individuals with DMD.

Neurogenesis is exclusive to two brain regions, one of which is the DG of the hippocampal formation (Kuhn et al., 2016), where newly born granule cells incorporate into established neuronal circuitry (Gage, 2000) and contribute to neuronal plasticity. Radial glia and a subpopulation of astrocytes act as stem cells that generate new glial cells and neurons in the adult hippocampal SGZ (Alvarez‐Buylla et al., 2001; Gebara et al., 2016; Kriegstein & Alvarez‐Buylla, 2009). Consistent with the known decrease in neural stem cells that occurs with age (Kuhn et al., 1996; Mathews et al., 2017; Yang et al., 2015), we observed decreased DG neurogenesis as the WT mice aged. However, in *mdx* mice, fewer newly born neurons were evident in very young mice (2 months old), and levels remained low at 4, 8 and 16 months. The functional importance of suppressed neurogenesis might be attributable to alterations in processes underpinning learning. Indeed, excitable immature hippocampal granule neurons, generated through neurogenesis, hold a strong propensity for long‐term potentiation (Ge et al., 2007), the proposed molecular mechanism by which activity‐dependent changes in pre‐ and/or postsynaptic signalling modify synaptic strength, which, in turn, underpins information storage (Bliss & Collingridge, 1993). Although not observed in all studies (Sesay et al., 1996), we (Stephenson et al., 2024) and others (Moore et al., 2002) have found that long‐term potentiation is suppressed in *mdx* hippocampal slices. As they mature, newly generated granule cells are capable of modulating long‐term potentiation (Aimone et al., 2009) and can impact on certain types of hippocampus‐dependent memory (Akers et al., 2014; Kitamura et al., 2009). However, unlike reports that focused on the CA1 hippocampal region (Miranda et al., 2016), no changes were observed in the density of granule cells in *mdx* DG regions, and numbers of these neurons remained stable throughout the life stages of *mdx* mice and WT mice.

Strong evidence has implicated the absence of full‐length Dp427 dystrophin from central neurons in the alteration of synaptic clustering of GABA_A_ receptors (Brunig et al., 2002; Knuesel et al., 1999; Miranda et al., 2009; Vaillend et al., 2010). This was linked to impaired ability to learn new tasks or store spatial memories (Chaussenot et al., 2015; Vaillend et al., 2004, 1995), which has been associated with age‐related deterioration of cognitive function (Bagdatlioglu et al., 2020). Inhibitory interneurons regulate the input of large principal cell populations and might specifically facilitate the consolidation of learning tasks (Shahidi et al., 2008). Enhancement of GABAergic regulation tends to impair the formation of new memories, whereas decreased GABA function can result in enhancement of memories (Brioni & McGaugh, 1988; Castellano & McGaugh, 1990; Jang et al., 2006; van der Linden et al., 1993). Increased numbers of a subset of GABAergic interneurons (fast‐spiking and basket cells immunopositive for parvalbumin) in the DG of 2‐ to 3‐month‐old *mdx* mice has been reported, possibly as a compensatory response to dysfunctional GABA_A_ receptors (del Tongo et al., 2009). However, in our studies, we noted that non‐principal cells immunopositive for GABA_A_ receptor, alpha 1 had large pyramidal‐shaped cell bodies. Mainly found in the hilus and granule cell layer, these are likely to be pyramidal basket cells (Ribak & Seress, 1983). We did not observe differences in the prevalence of these GABA_A_ receptor‐expressing cells between mouse strains at different age points, similar to reports by others (Morley, 2007). Given that dystrophin is normally co‐expressed with GABA_A_ receptors on the postsynaptic density and loss of dystrophin reduces the number and size of inhibitory synapses (Knuesel et al., 1999), the regulatory actions of these neurons in functions such as the encoding and consolidation of memories is likely to be modified. Thus, the impact of dystrophin loss on hippocampal function is likely to relate to altered regulatory function in GABAergic neurons, rather than changes in cellular density per se.

Given their role in facilitating memory‐consolidating signalling pathways, it is no surprise that much research has been focused on neuronal circuitry in the hippocampus, which can be modified by loss of both dystrophin and utrophin (Knuesel et al., 2001), although utrophin does not appear to be promising in addressing cognitive deficits (Perronnet et al., 2012). However, less attention has been given to astrocytes, the most abundant cell type in the CNS. Critical for normal hippocampal function by supporting neuronal nutrition, buffering ions and mediating the recycling of neurotransmitters such as glutamate, GABA and glycine (Verkhratsky & Nedergaard, 2018), astrocytes are more than simply support cells. They participate in bidirectional communication with neurons and have important functions in the pathophysiology of neurological diseases (Ricci et al., 2009). Dp71 is predominantly expressed in astrocytes (Hendriksen et al., 2016; Patel et al., 2019), and *Dp71* null mice had reduced density of astrocytes with abnormal morphology (Giocanti‐Auregan et al., 2016). Moreover, *Dp71* knockout mice exhibit abnormal social exploratory behaviour and learning of aversive cue–outcome associations, further implicating this isoform, and the cells in which it is expressed, in cognitive dysfunction (Miranda et al., 2024).

In our study, variability in the density of WT astrocytes was noted. Nonetheless, age‐related astrogenesis was clearly evident. In contrast, a striking reduction in numbers of astrocytes was apparent from 4 months onwards. This aligns with reports on human DMD induced pluripotent stem cell‐derived astrocytes, which are more susceptible to injury and cell death, resulting in reduced numbers (Lange et al., 2022). Additionally, astrocyte morphology is abnormal in *Dp71* null mice (Giocanti‐Auregan et al., 2016). However, in *mdx* mice, which expresses a mutation that blocks *Dp427* transcription and translation, astrocyte loss was the most striking change in the DG. Thus, loss of neuronal dystrophin might impact the density of DG astrocytes. Alternatively, general peripheral and/or central inflammation associated with dystrophinopathy might underpin this change in astrocyte density. The specific mechanisms remain to be elucidated, but the hippocampus is particularly vulnerable to upregulation of reactive genes in astrocytes (Clarke et al., 2018), which become sensitized with age and are associated with inflammation.

Astrocytes exert functions that are specific to the layer in which they reside and reflect the neuronal components that are present in that hippocampal region (Chalmers et al., 2024; Karpf et al., 2022). In the neurogenic niche, the highly vascularized micro‐environment that contains neural progenitor cells (Cole et al., 2022; Stolp & Molnar, 2015), dystrophin‐expressing astrocytes (Garcia‐Cruz et al., 2023) underpin neurogenesis (Shapiro et al., 2005) and hippocampal plasticity (Kempermann et al., 2015). GFAP‐expressing radial glia‐like astrocytes cradle neurogenically derived neurons in the SGZ and create a scaffold for the outgrowth of neuronal processes (Shapiro et al., 2005). No differences in the number of astrocytes in the SGZ, which tended to be small and stretched along the border of the cell body layer (Chalmers et al., 2024), were noted between WT and *mdx* mice at any age point. However, given that astrocytes generated from DMD‐derived induced pluripotent stem cells exhibited cytoskeletal abnormalities (Lange et al., 2022) and defective Ca^2+^ homeostasis, which impacted neuronal cell function (Patel et al., 2019), changes related to dysfunctional dystrophic astrocytes in this region of the DG require further exploration. In the hilus region, the number of astrocytes, which tended to have a bushy cytoarchitecture, varied with age, but despite suppression of astrocyte numbers at 8 months in *mdx* mice, across all age points the loss of dystrophin did not result in significant differences in the density of astrocytes in this region. In the ML, where the dendritic branches of the granule cell bodies interact with perforant path fibres, there was a clear impact of age on astrocyte numbers. Moreover, numbers of astrocytes were notably decreased in *mdx* mice. Astrocytes in this region, which tend to create strongly coupled syncytia and exhibit large dendritic trees, consistent with interaction with many synapses (Chalmers et al., 2024; Karpf et al., 2022), have been recognized in the integration of newly born neurons into existing neural circuitries (Krzisch et al., 2015). Thus, decreased neurogenesis early in the life of *mdx* mice, in addition to life‐long reductions in ML astrocytes that integrate adult‐born neurons into neural circuits and facilitate the consolidation of new memories, might contribute to deficits in hippocampal function and contribute to the cognitive deficits described in this mouse model (Chaussenot et al., 2015; Moore et al., 2002; Vaillend et al., 2004, 1995).

Finally, we examined the resident brain immune cells, microglia, which have important functions in normal CNS homeostasis and during inflammation. Surveillant microglia are proposed to regulate the fate and development of adult‐born neurons, regulating the number that survive to become mature neurons and fine‐tuning existing neural circuits by engulfing weaker synapses (Luo et al., 2016). Ramified microglia, which actively screen the brain and establish neuronal contacts, thereby modulating neuronal activity, were observed at comparable densities in WT and *mdx* 2‐month‐old mice. Although dystrophins are virtually absent from microglial cells in mouse brains (Garcia‐Cruz et al., 2023), the number of microglia with morphology consistent with active phenotype, which confers capabilities for phagocytosis and inflammatory cytokine release, was increased in the DG of 2‐month‐old *mdx* mice. This might reflect the pro‐inflammatory nature of dystrophinopathies. Mice aged >4 months primarily expressed microglia with active morphologies, which aligns with the concept that microglia become more reactive with age (Mosher & Wyss‐Coray, 2014), but no difference was detected between strains at the later age points.

## 5 CONCLUSIONS

In the context of reported decreases in the volume of hippocampal grey matter and enlarged lateral ventricles, particularly from 12 months onwards, in dystrophin‐deficient *mdx* mice (Bagdatlioglu et al., 2020), we have explored the cellular composition of the dystrophic hippocampal DG at four time points over the lifespan of the mice. In young *mdx* mice, early in disease development, we observed suppressed neurogenesis, which might have an acute impact on the formation of new memories but could also contribute to changes in the density of neurons and glial cells over time. That said, no changes were detected in the numbers of excitatory dentate granule cells, which are strategically situated to regulate the flow of information into the hippocampus, nor in the numbers of GABA_A_ receptor, alpha 1‐expressing non‐principal cells, although these observations imply nothing regarding changes in the functional properties of these cells. The most striking differences detected were in astrocyte numbers. Astrogenesis results in increased numbers of astrocytes over time, but this was suppressed in dystrophic animals, and our findings suggest that this relates primarily to astrocytes in the molecular layer. Functionally, astrocyte activity evolves from being generally protective and critical for normal neuronal function early in life, and this function might be lacking in hippocampal dystrophin‐deficient tissue. However, in the course of ageing, astrocytes can become sensitized and are associated with inflammation. Additional studies are needed to further our understanding of how the age‐related changes in dystrophic hippocampal cellular composition are related to functional cognitive deficits and behavioural changes.

## AUTHOR CONTRIBUTIONS

Kimberley A. Stephenson and Polly Peters were involved in the acquisition, analysis and interpretation of the data presented in this manuscript. Mark G. Rae and Dervla O'Malley conceived and designed the experimental work and contributed to the interpretation of the findings. All authors were involved in drafting the work and critically reviewing it for intellectual content. All authors approved the final version of the manuscript and agree to be accountable for all aspects of the work in ensuring that questions related to the accuracy or integrity of any part of the work are appropriately investigated and resolved. All persons designated as authors qualify for authorship, and all those who qualify for authorship are listed.

## CONFLICT OF INTEREST

None declared.

## Data Availability

Data are available upon request.
